# Is there a difference in response rate and degree of satisfaction among family members of survivors and nonsurvivors at admission to intensive care?

**DOI:** 10.1186/cc9946

**Published:** 2011-03-11

**Authors:** C Teixeira, J Keating, I Silva, A Veloso, O Ribeiro, I Aragão

**Affiliations:** 1 Centro Hospitalar do Porto, Portugal; 2 Universidade do Minho, Braga, Portugal; 3 Faculdade de Medicina, S. Bioestatística e Informática Médica, Porto, Portugal

## Introduction

The objective was to identify whether there are differences in the degree of satisfaction among family members of survivors and nonsurvivors on admission to the ICU. Also, to identify who they are and what are the sociodemographic characteristics of the relatives answering a questionnaire for assessing the needs of relatives of patients admitted to our ICU, and what are the clinical and sociodemographic characteristics of patients.

## Methods

A letter was sent to all families who had a relative in the ICU in the period of 1 year, with a sealed envelope with the address, and the questionnaire: characterization of the family, assessing the satisfaction of the needs in family-gathering areas for support, comfort, access, information, and trust. We also collected sociodemographic data and patient records. Statistical analysis was performed using SPSS v.17.0.

## Results

We obtained responses from 90 families, 43% spouses, mean age 47 years, 65% female. Characterization of patients (median (P25 to P75)): 78% male, age 60 years (41 to 73), SAPS 43 (33 to 54), number of hospital days in the ICU 9 days (4 to 16). We obtained a higher and statistically significant (*P *< 0.05) response rate in relatives of patients hospitalized longer and in those who survived. The family satisfaction was generally good. The relatives of the survivors were more satisfied in all dimensions evaluated (a value closer to greater satisfaction), although this difference was statistically significant only in the comfort dimension (*P *= 0.003).

## Conclusions

Contrary to other studies we found that relatives of the survivors are more satisfied with most aspects of care received, better meeting their needs than family members of nonsurvivors, although this difference is statistically significant only in the dimension comfort. The results emphasize the need for improved measures of comfort in the ICU. One factor, among others, to explain this result may be that on one hand the aspects of patient-centered care and family were similar in both groups, but on the other hand the relatives of the survivors feel more a lack of space suitable for families that currently do not exist in our ICU. There is a growing recognition that families are an integral part of the modern ICU and that we should incorporate the findings of this evaluation of needs and family satisfaction in quality improvement in the ICU.

